# Prevalence of amyotrophic lateral sclerosis in the United States using established and novel methodologies, 2017

**DOI:** 10.1080/21678421.2022.2059380

**Published:** 2022-04-15

**Authors:** PAUL MEHTA, JAIME RAYMOND, RESHMA PUNJANI, MOON HAN, THEODORE LARSON, WENDY KAYE, LORENE M. NELSON, BARBARA TOPOL, OLEG MURAVOV, CORINA GENSON, D. KEVIN HORTON

**Affiliations:** 1 Centers for Disease Control and Prevention/Agency for Toxic Substances and Disease Registry, National ALS Registry (CDC/ATSDR), Atlanta, GA, USA,; 2 McKing Consulting Corporation, Atlanta, GA, USA; 3 Department of Epidemiology and Population Health, Stanford University School of Medicine, Stanford, CA, USA

**Keywords:** Amyotrophic lateral sclerosis, prevalence, United States, 2017, capture–recapture, epidemiology, risk, models

## Abstract

**Objective:**

To estimate the prevalence of amyotrophic lateral sclerosis (ALS) in the United States for 2017 using data from the National ALS Registry (Registry) as well as capture–recapture methodology to account for under-ascertainment. Established in 2010, the Registry collects and examines data on ALS patients in the US to better describe the epidemiology of ALS (i.e. risk factor exposures, demographics).

**Methods:**

The Registry compiled data from national administrative databases (from the Centers for Medicare and Medicaid Services, the Veterans Health Administration, and the Veterans Benefits Administration) and a voluntary enrollment data through a web portal (www.cdc.gov/als). To estimate the number of missing cases, capture–recapture methodology was utilized.

**Results:**

The Registry conservatively identified 17,800 adult persons (lower-bound estimate) who met the Registry definition of ALS for an age-adjusted prevalence of 5.5 per 100,000 US population. Using capture–recapture methodology, we obtained a “mean case count” of 24,821 ALS cases (prevalence of 7.7 per 100,000 U.S. population) and estimated the upper-bound estimate to be 31,843 cases (prevalence of 9.9 per 100,000 U.S. population). The pattern of patient characteristics (e.g. age, sex, and race/ethnicity) remained unchanged from previous Registry reports. Overall, ALS was most common among whites, males, and persons aged 60–69 years. The age groups with the lowest number of cases were persons aged 18–39 years. Males had a higher prevalence than females overall and across all data sources.

**Conclusions:**

Existing Registry methodology, along with capture-recapture methodology, are being used to better describe the epidemiology and demographics of ALS in the US.

## Introduction

Amyotrophic lateral sclerosis (ALS), commonly known as Lou Gehrig’s disease, is a progressive and fatal neuromuscular disease with the majority of ALS patients dying within 2–5 years of receiving a diagnosis (1,2). Familial ALS, a hereditary form of the disease, accounts for 5–10% of cases, whereas the remaining cases (sporadic ALS) have no clearly defined etiology (3,4). ALS affects persons of all races and ethnicities; however, whites, males, non-Hispanics, persons aged ≥60 years, and those with a family history of ALS are more likely to develop the disease (5–10). No cure for ALS has yet been identified, and the lack of proven and effective therapeutic interventions is an ongoing challenge. Treatments currently available, Edaravone and Riluzole, do not cure ALS, but slow disease progression in certain patients (11,12).

Potential risk factors for ALS have been identified such as exposures to heavy metals, pesticides, ß-*N*-methylamino-l-alanine (BMAA), military service, and trauma among others (13–18). The role of environmental risk factors with ALS remains an active topic area of investigation (14,16,19–21).

In 2008, the U.S. Congress passed the ALS Registry Act, authorizing the creation of the National ALS Registry (Registry) by the Centers for Disease Control and Prevention’s (CDC) Agency for Toxic Substances and Disease Registry (ATSDR). The objectives of the Registry include describing the incidence and prevalence of ALS, examining risk factors such as environmental and occupational factors, and characterizing the demographics of persons living with ALS (22).

Nationally notifiable diseases and conditions, primarily infectious in nature, are reported to the CDC on an annual basis (23). ALS, like most noncommunicable diseases apart from cancer, is not a reportable disease at the local or state levels (except for the state of Massachusetts) nor is it notifiable to federal health agencies such as the CDC/ATSDR (24).

Here, we calculate the 2017 prevalence of ALS in the adult (≥18) US population using the National ALS Registry self-enrollment portal, the national administrative databases, and capture–recapture methodology for the evaluation of missing cases.

## Methods

### Established and validated algorithm for identifying ALS cases

The National ALS Registry uses a two-pronged approach to identify prevalent cases of ALS in the US. The first approach identifies cases from three large national administrative databases (Medicare, Veterans Health Administration, and Veterans Benefits Administration) by using an algorithm with elements such as the International Classification of Diseases (ICD) 10th revision code for ALS, frequency of visits to a neurologist, cause of death via national death certificate data, and prescription drug use (25). A pilot tested algorithm is applied to the administrative data that identifies persons with ALS on the basis of encounter codes such as having ALS listed as a code in the visit record or having such a code and having seen a neurologist, a death certificate listing ALS as a cause or contributing cause of death, and prescription for Riluzole (9). If the patient meets the criteria, e.g. a person aged <65 years with an encounter coded for ALS in Medicare and a neurologist visit, the patient is identified as a “definite ALS case.” The Registry categorizes an ALS case as “definite ALS,” “possible ALS,” and “not ALS”. Only “definite ALS” cases are entered into the Registry.

Beginning in 2015, the Registry initiated use of Medicare Part C data (Medicare Advantage Plan) as an additional data source using the same algorithm as is applied to Medicare Fee for Service data (Medicare Parts A, B, D). Medicare Advantage is administered by private insurance companies who are contracted by Medicare. Cases identified as “definite ALS” in 2015 from Medicare Advantage were carried over into 2016 and, if they were alive and ascertained in that data source again in 2016, were eligible to meet the criteria to be included as definite cases of ALS. Medicare Advantage data for 2016 and 2017 have been requested but not received as of the time of this publication. Cases determined to be “definite ALS” cases will be added to future analyses.

The second approach is a secure web portal that enables persons with ALS to enroll in the Registry, thereby enabling the identification of additional cases not recorded in the national administrative databases. Cases from both sources are then merged and deduplicated. Once an ALS case is identified, the patient remains a case until confirmed deceased through the CDC’s National Death Index. This is referred to as cumulative prevalence of ALS and is calculated by using the deduplicated total number of persons with ALS identified through the two-pronged approach for the numerator. The 2017 US Census estimate is used for the denominator and 95% confidence intervals are calculated (26). This method is referred to as the established or original algorithm for calculating national prevalence estimates.

### Capture–recapture methodology for identifying missing ALS cases

Because ALS is not a notifiable condition, under-ascertainment of cases invariably occurs. However, statistical approaches are now being used to address missing cases. Capture–recapture is a widely used statistical technique that examines the overlap in identification of cases from data sources and uses this information to estimate the number of cases who were not identified by any of the sources, thus enabling a conclusion about the completeness of ascertainment (27). Capture–recapture method has also been used by other studies across different race and ethnic background to correct for the missing cases in the estimations of incidence rates (28–30). For the purposes of estimating the degree of under-ascertainment by the Registry data source, Nelson et al. applied capture–recapture methods to the 15,927 cases identified by the three data sources during the registry year 2014 (31). This estimated the number of missing ALS cases to be 12,578, resulting in an under-ascertainment-corrected ALS case count of 28,505. The percent of the total missed by the three data sources was 12,578/28,505 or 44.1%, which we apply here to 2017 Registry data (31). We used the same overall estimate of the percentage of missing cases for the 2017 Registry data by considering the observed number of cases in 2017 (*n* = 17,800) as comprising only 56% of the total cases, yielding a capture–recapture estimated total case count of 31,843 (i.e. 44% or 14,043/31,843 were missing). Previous years of prevalence data from 2014 to 2016 were also reported and adjusted in these analyses. For our final estimate of ALS prevalence in 2017, we chose an estimate that was at the midpoint (or mean) of the observed case count and the capture-recapture estimated total number of cases (i.e. 24,821 which is the mean of 17,800 and 31,843). We reasoned that this approach was more conservative than relying on the capture–recapture corrected total estimate as that estimate represents an upper bound and the unadjusted total represents a lower bound of the “true” US prevalence. Similar adjustments for strata-specific under-ascertainment were applied to levels of gender, race, and age-category.

## Results

For 2017, the National ALS Registry found 17,800 persons having definite ALS with a prevalence of 5.5 per 100,000 persons by applying the algorithm to possible cases identified by the national administrative databases and the web portal (Table 1). Persons aged 18–39 years had the lowest prevalence (0.6 cases per 100,000), and persons aged 70–79 had the highest (19.5 per 100,000, Table 1). As in all previous analyses conducted by the Registry, the prevalence in males (7.0 cases per 100,000 population) was higher than that in females (4.1) (5–9). The prevalence in whites (5.5 cases per 100,000 population) was almost twice that in Blacks (2.8 per 100,000, Table 1).

For 2017, to account for under-ascertainment, we used the estimate of percentage missing (44%) that we had previously estimated in the 2014 capture-recapture analyses (31). Using 2017 registry data, this means that the observed number of cases (*n* = 17,800) comprised only 56% (100–44%) of the total cases, yielding a capture–recapture estimated total case count of 31,843 and the estimated number of missing cases of 14,043 (i.e. 31,843 minus 17,800). The corresponding adjusted mean prevalence was 7.7 per 100,000 population (Table 1). Persons aged 18–39 years had the lowest prevalence (1.2 cases per 100,000), and persons aged 70–79 had the highest prevalence (29.8 per 100,000), as was observed in previous registry years. The percentage missing for <65 age group was 51.6% and 34.8% for those over the age of 65.

**Figure 1.**
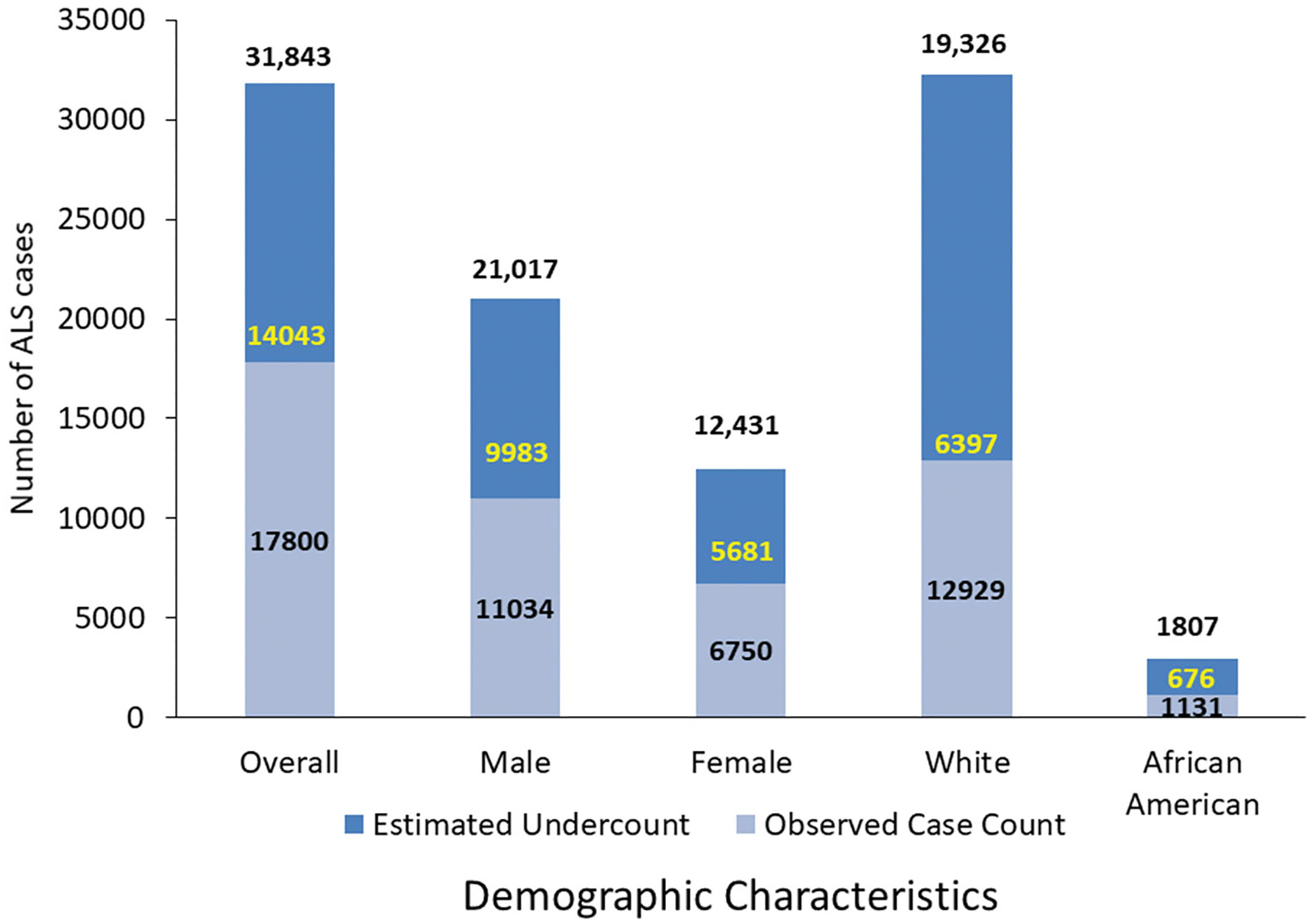
Number of ALS cases by sex, race, and overall, adjusted for case undercount using the capture-recapture methodology and missing case estimates – National ALS Registry, United States, 2017.

Males had a higher mean prevalence rate of 9.8 per 100,000 than females (5.9 per 100,000). We applied the sex-specific estimates of % missing from the Nelson et al. report and estimated that 9983 males cases (47.5%) were missing and 5681 females (45.7%) were missing (31) (Figure 1). The mean prevalence in Whites (6.9 cases per 100,000 population) was higher than in Blacks (3.6 per 100,000, Table 1). The percentage of missing cases for Blacks was slightly higher than that of Whites, 37% versus 33%.

The Registry has previously published case counts and prevalence rates for 2014–2016 which showed prevalence rates between 5.0 (2014) and 5.2 (2015, 2016) per 100,000 cases. A corrective mean prevalence was as follows per 100,000 cases: 7.0 (2014), 7.2 (2015), and 7.1 (2016) (Table 2).

## Discussion

This report presents updated ALS prevalence estimates for the US using an established case-ascertainment methodology and capture–recapture methodology to adjust for under-ascertainment. The Registry’s case ascertainment methodology has been used since the first national ALS prevalence estimate for 2010–2011, released in July 2014, and for all successive prevalence reports (5). For the 2017 capture–recapture estimate, we used log-linear modeling to estimate the missing number of ALS cases in the US and to provide an under-ascertainment- adjusted estimate of ALS prevalence (31). For discussion purposes, the authors will focus on the mean prevalence estimates and not the upper bound estimates.

In the US, ALS patients have a wide choice of healthcare options such as Medicare, which covers Part A (covers hospital costs) and Part B (covers doctor and outpatient care); Medicare Advantage, Part C, offered by private insurance carriers approved by Medicare; and Part D which assists in paying for prescription drugs (32). Part C data were not available from Medicare for these analyses. ALS patients who have served in the military can also seek care through the Veterans Administration (VA) as well as any of the Medicare options described above (33). Moreover, the adjustment of case counts which can lead to a decrease or increase of estimates is not uncommon in public health especially when new methodologies are used to measure disease burden more accurately (34,35). While every effort has been made to determine case counts of ALS in the US, it is not possible to ascertain all cases when data are fragmented, and disease notification is not required nationally.

This report does not use Medicaid data because reporting requirements differ by each state and data are not yet available from all states for 2017. In addition, because Medicaid is need-based, it is estimated the minimal number of ALS cases identified from Medicaid is unlikely to have a noticeable effect on the prevalence estimates. Nevertheless, the Registry has requested Medicaid data for 2016–2018 and eligible cases will be added to successive analyses when available.

### Interpretation of prevalence estimates

The utilization of two methodologies (i.e. Registry and capture–recapture) provides a comprehensive approach for estimating national ALS prevalence trends and establishing lower, mean, and upper bound estimates. For 2017, 17,800 patients were identified as definite ALS cases. These cases represent a lower bound estimate of the number of cases in the US. Conversely, the 31,843 cases estimated using capture–recapture statistics can be viewed as an upper bound estimate. Establishing an upper and lower limit allows a better estimation of variability. The mean value of 24,821 or prevalence of 7.7 per 100,000 population is likely a better representation of actual ALS cases in the US (Figure 1). Moreover, Kaye et al. previously evaluated the completeness of the Registry and found the Registry was missing 43% of the cases found in surveys of state and metropolitan areas (36). That finding is consistent with the capture–recapture estimate of 44% missing in these analyses.

**Figure 2.**
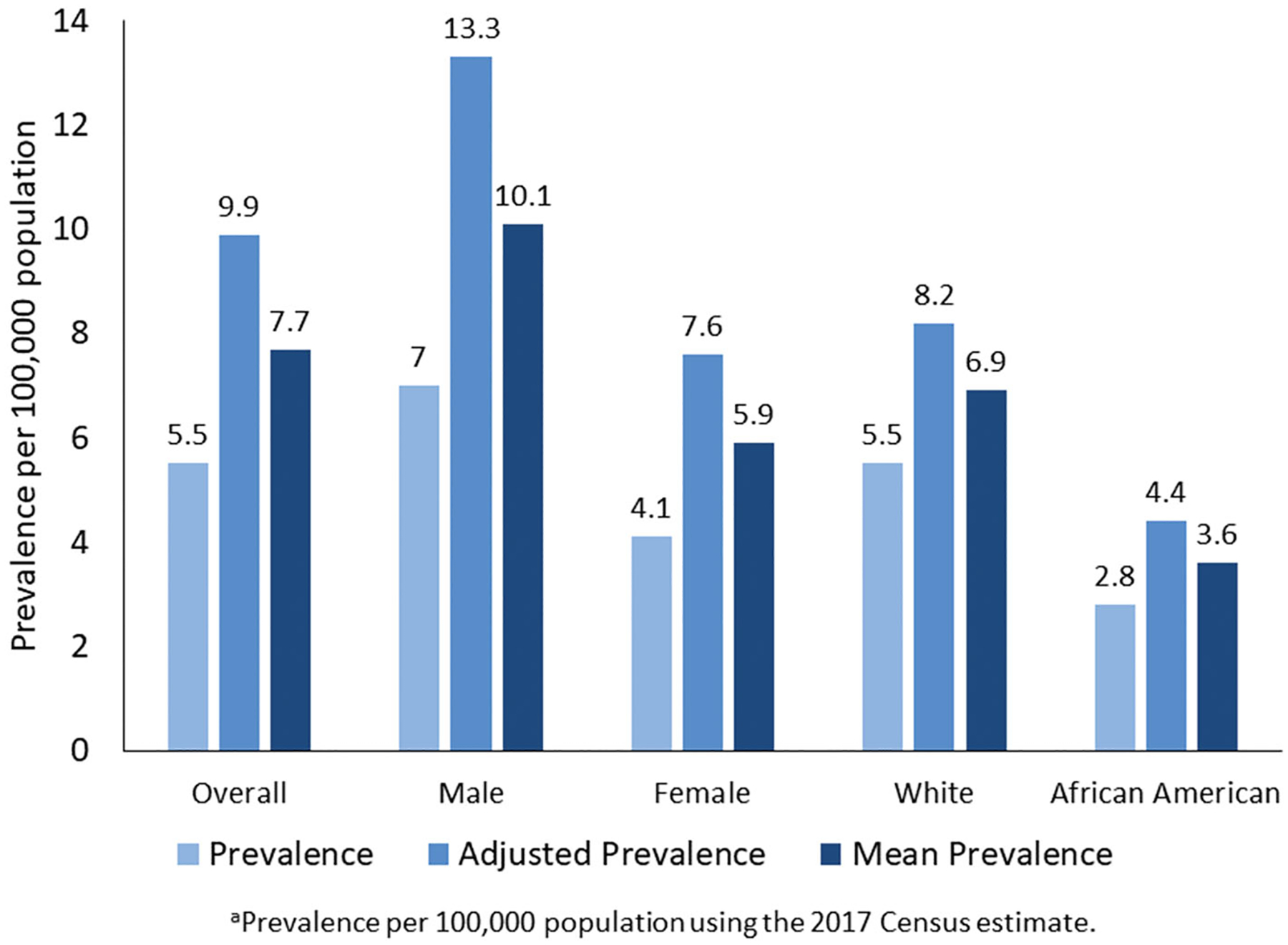
Estimated prevalence, adjusted prevalence, and mean prevalence per 100,000 of amyotrophic lateral sclerosis by sex, race, and overall – National ALS Registry, United States, 2017^a^. Prevalence (light blue) is the estimation without the application of capture–recapture method. Adjusted prevalence (blue) is the upper-bound estimate using capture–recapture method. Mean prevalence (dark blue) is the midpoint estimation between the established algorithm and the estimation obtained by capture–recapture methods.

In 2018, the ALS Association (ALSA) served 20,101 patients at its chapters across the US (33). Other groups such as the Muscular Dystrophy Association (MDA) and the Les Turner ALS Foundation also served ALS patients across the country with an overlap of patient care at clinics that are jointly run by ALSA and MDA (37). Thus, the mean case count of 24,821 is further supported as not all ALS patients will be served by patient care organizations and overlaps are also possible. When evaluating gender, males continue to have a higher mean prevalence than females (Figure 2). ALS impacts males at a much higher rate than females and this is not unexpected. Patients <59 also have a higher degree of under-ascertainment, most likely as a result of older patients remaining on their private insurance (Table 1).

Capture–recapture also estimated approximately 1807 cases in Blacks from 1131 cases which were found using the established algorithm or a net gain of 633 cases. Data for Hispanics are not available from the administrative datasets because these cases are classified as “Other.” Findings from both methods demonstrate that ALS continues to impact Whites, especially males, more so than any other group (Figure 2).

The adjustment of prior years, 2014–2016, showed a rate increase of 2.0 for 2014 and 2015 and 1.9 for 2016. The mean prevalence for these years was between 7.0 and 7.2 per 100,000 cases (Table 2). While 2017 demonstrated a higher mean prevalence rate of 7.7, this was due to better case-ascertainment by the Registry and not necessarily an upward trend in national prevalence. Additional years of data are needed to determine trends. As ALS patients continue to receive comprehensive multi-disciplinary care, an increase in prevalence may occur (38–40). Though without new therapeutic options, the contribution of slowed disease progression to increased or sustained prevalence may still be some years away.

### Surveillance challenges

It is unknown what percentage of ALS patients seek care from private insurance companies and what percentage will eventually migrate to either Medicare options (Part A/B or Part C), VA care, or a combination of the choices stated above. At the time of their initial diagnosis, many ALS patients are covered by employer-provided private insurance. Patients who are insured through an employer-sponsored healthcare plan may chose to remain on their plan indefinitely. However, if identifiable patient data were available from private health insurance providers, a combination of both private and public insurance medical claims could theoretically be used to identify all patients with ALS in the US. These providers include preferred-provider organizations (PPO), health maintenance organizations (HMO), high-deductible health plans (HDHP), point of service (POS), and exclusive provider organizations (EPO). As of 2017, there were 907 health insurance companies in the US and its territories (41).

Patients may also seek to get approved for Social Security Disability Insurance (SSDI) and eventually Medicare (42). In addition, patients who are enrolled in Medicare may not be identified by the Registry if they do not meet the prerequisites of the algorithm. Patients who have served in the military are eligible for both Medicare and VA benefits. It is believed most cases missed by all methods, and as estimated by capture-recapture, are patients who receive care outside of the Medicare and Veteran Administration health systems and who are covered either by private insurance or, to a much lesser degree, by Medicaid. Insurance claims data are available from a number of different systems such as Optum Health Services, Truven MarketScan, and IQVIA, but a major limitation is the unavailability of personally identifiable information (PII) (43–45). Without PII such as name, date of birth, age, or sex, the Registry is unable to match cases from private insurance with national administrative datasets. Furthermore, due to the variability and fragmented health care delivery model in the US, determining actual case counts is not possible as it is with ALS registries in Europe (countries with a single-payer health care system) (46). In addition, ALS variability from patient to patient must be mentioned as some patients may rapidly succumb to the disease and may never transition to Medicare or the VA systems. This may include ALS patients with fast disease progression, short diagnostic delay, bulbar onset, or lower ALS Functional Rating Scale – Revised (ALSFRS-R) when compared with slow progressing patients (47,48).

Of note, to estimate disease burden nationally, incidence and prevalence data for communicable diseases, other reportable/notifiable conditions, and cancer are generally more robust, timely, and accurate than those for non-notifiable chronic conditions. Though, as with any surveillance system, cases may also be missed or underreported for some communicable diseases such as tuberculosis, human immunodeficiency virus (HIV), and others (49,50).

### Future directions

For future national prevalence estimates, the Registry is reviewing its case-ascertainment algorithm to better identify ALS cases. The current established algorithm has been used for all prior prevalence reports since calendar year 2010 and a review to determine whether algorithm modification is necessary is underway. If a change is warranted, a newly modified algorithm will be applied to future analyses.

To better assess the number of missing patients, the Registry is seeking to add new data sources, including sources such as new or existing state-based registries as well as cases from the above-mentioned ALS patient organizations. In addition, the Registry will seek to obtain cases from private insurance databases. Barriers such as patient consent will also need to be addressed prior to receiving data.

The pandemic which started in 2019 has impacted outreach to patients by the Registry and its partners (ALS Association, Muscular Dystrophy Association, and Les Tuner ALS Foundation). This has been observed in the online self-portal (data not shown). When pandemic-associated restrictions are lifted such as face-to-face patient interactions, the Registry intends to work closely with its partners to target areas with higher minority populations such as California, Texas, Florida, and New York.

## Limitations

The findings in this report are subject to at least three limitations. First, because ALS continues to be a non-notifiable disease, it is challenging to ensure that all newly diagnosed and prevalent ALS cases in the United States are captured in the Registry and, therefore, the possibility of under-ascertainment exists. Even with notifiable conditions such as communicable infections, under-ascertainment exists and, in general, even the best surveillance system will not be able to identify all cases. Second, although every attempt was made to de-duplicate the files using the established algorithm, differences in fields collected by the different sources, misspellings of names, and data entry errors could have prevented records from merging correctly. However, it is unlikely that this occurred in numbers sufficient to affect the overall conclusions or in a differential manner that affected conclusions. Finally, without personally identifiable information including name, date of birth, age, or sex, the Registry is currently unable to match cases from private insurance with national administrative datasets.

## Conclusions

The establishment of the National ALS Registry fills an important scientific gap by providing estimates of incidence, mortality, and prevalence of this disease and facilitates further study of risk factors and etiology. Existing Registry methodology, along with capture–recapture methodology, are being used to better describe the epidemiology and demographics of ALS in the US. While 2017 demonstrated a higher mean prevalence rate of 7.7, this was due to better case-ascertainment by the Registry and not necessarily an upward trend in national prevalence. We continue to improve the Registry and add enhancements to better ascertain ALS cases by evaluating the established algorithm for any needed updates or changes as well as evaluating new data sources. CDC/ATSDR is committed to monitoring trends of ALS prevalence in the United States and advancing ALS research.

## Figures and Tables

**Table 1.** Number and percentage of amyotrophic lateral sclerosis (ALS) cases and estimated prevalence, by age group, sex, and race – National ALS Registry, United States, 2017.

		Established algorithm			Capture-recapture methods				Mean estimation	
Characteristic	Population^a^	No. (%) cases	Prevalence (95% CI)^b^	Observed count	Reported % missing^c^	Missing cell estimate^c^	No. cases, adjusted^d^	Prevalence^b^,^d^	No. cases^e^	Prevalence^b,e^
Total	321,004,407	17,800 (100.0)	5.5 (5.4, 5.6)	17,800	44.1%	14,043	31,843	9.9	24,821	7.7
Age groups (years)										
18–39	92,704,957	518 (2.9)	0.6 (0.56, 0.64)	518	51.6%	552	1070	1.2	794	0.9
40–49	40,476,486	1471 (8.3)	3.6 (3.3, 3.8)	1471	51.6%	1568	3039	7.5	2255	5.6
50–59	43,278,449	3339 (18.3)	7.7 (7.3, 8.1)	3339	51.6%	3560	6899	15.9	5119	11.8
60–69	36,721,684	4814 (27.0)	13.1 (12.7, 13.5)	4814	43.2%	3661	8475	23.1	6645	18.1
70–79	21,533,719	4189 (23.5)	19.5 (19.0, 20.0)	4189	34.8%	2236	6425	29.8	5307	24.6
≥80	12,426,454	1428 (8.0)	11.5 (11.1, 11.9)	1428	34.8%	762	2190	17.6	1809	14.6
Unknown	–	2041 (11.5)								
Sex										
Male	158,018,753	11,034 (62.0)	7.0 (6.9, 7.1)	11,034	47.5%	9983	21,017	13.3	16,026	10.1
Female	162,985,654	6750 (37.9)	4.1 (4.0, 4.2)	6750	45.7%	5681	12,431	7.6	9590	5.9
Unknown	–	16 (0.1)								
Race										
White	234,370,202	12,929 (72.6)	5.5 (5.4, 5.6)	12,929	33.1%	6397	19,326	8.2	16,127	6.9
Black	40,610,815	1131 (6.4)	2.8 (2.7, 2.9)	1131	37.4%	676	1807	4.4	1469	3.6
Other	–	973 (5.5)		973	37.4%	1554	2527			
Unknown		2511 (17.9)		2511	75%	10,166	12,677			

a Population based on US Census 2017.

b Cases per 100,000 US population.

c Estimation based on capture–recapture methodology (31).

d Adjusted estimation using the previously reported capture–recapture result.

e Mean or midpoint estimation between the established algorithm and the estimation obtained by capture–recapture methods.

**Table 2.** Number and percentage of amyotrophic lateral sclerosis (ALS) cases and estimated prevalence – National ALS Registry, United States, 2010–2016.

	Established algorithm		Capture-recapture method			Mean estimation	
Year	No. cases/observed count	Prevalence^a^	No. cases, missing^b^	No. cases, corrected^b^	Prevalence^a^, corrected^b^	No. cases^c^	Prevalence^a,c^
2010–2011	12,187	3.9	9614	21,801	7.0	16,994	5.5
2012	14,713	4.7	11,607	26,320	8.4	20,517	6.5
2013	15,908	5.0	12,550	28,458	9.0	22,183	7.0
2014	15,927	5.0	12,565	28,492	8.9	22,209	7.0
2015	16,583	5.2	13,082	29,665	9.2	23,124	7.2
2016	16,424	5.2	12,957	29,381	9.1	22,903	7.1

a Cases per 100,000 population.

b Estimation based on capture–recapture methodology.

c Mean or midpoint estimation between the established algorithm and the estimation obtained by capture–recapture methods.
